# Zoonotic tuberculosis due to Mycobacterium bovis in developing countries.

**DOI:** 10.3201/eid0401.980108

**Published:** 1998

**Authors:** O Cosivi, J M Grange, C J Daborn, M C Raviglione, T Fujikura, D Cousins, R A Robinson, H F Huchzermeyer, I de Kantor, F X Meslin

**Affiliations:** World Health Organization, Geneva, Switzerland. cosivio@who.ch

## Abstract

The World Health Organization (WHO) estimates that human tuberculosis (TB) incidence and deaths for 1990 to 1999 will be 88 million and 30 million, respectively, with most cases in developing countries. Zoonotic TB (caused by Mycobacterium bovis) is present in animals in most developing countries where surveillance and control activities are often inadequate or unavailable; therefore, many epidemiologic and public health aspects of infection remain largely unknown. We review available information on zoonotic TB in developing countries, analyze risk factors that may play a role in the disease, review recent WHO activities, and recommend actions to assess the magnitude of the problem and control the disease in humans and animals.


### Zoonotic Tuberculosis due to *Mycobacterium bovis* in Developing Countries

O. Cosivi,* J.M. Grange,† C.J. Daborn,‡ M.C. Raviglione,* T. Fujikura,¶
D. Cousins,§ R.A. Robinson,** H.F.A.K. Huchzermeyer,††
I. de Kantor,‡‡ and F.-X. Meslin*
*World Health Organization, Geneva, Switzerland; †Imperial College School of Medicine, National Heart and Lung Institute, London, United Kingdom; ‡Edinburgh University, Edinburgh, Scotland; ¶The University of Zambia, Lusaka, Zambia; §Department of Agriculture, South Perth, Australia; **College of Veterinary Medicine, Veterinary Teaching Hospital, St. Paul, Minnesota, USA; ††Onderstepoort Veterinary Institute, Onderstepoort, South Africa; and ‡‡Pan American Health Organization/ World Health Organization, Buenos Aires, Argentina

The World Health Organization (WHO) estimates that human tuberculosis (TB) incidence and deaths for 1990 to 1999 will be 88 million and 30 million, respectively, with most cases in developing countries. Zoonotic TB (caused by *Mycobacterium bovis)* is present in animals in most developing countries where surveillance and control activities are often inadequate or unavailable; therefore, many epidemiologic and public health aspects of infection remain largely unknown. We review available information on zoonotic TB in developing countries, analyze risk factors that may play a role in the disease, review recent WHO activities, and recommend actions to assess the magnitude of the problem and control the disease in humans and animals.

Tuberculosis (TB), one of the most widespread infectious diseases, is the leading cause of death due to a single infectious agent among adults in the world. *Mycobacterium tuberculosis* is the most common cause of human TB, but an unknown proportion of cases are due to *M. bovis* (1). In industrialized countries, animal TB control and elimination programs, together with milk pasteurization, have drastically reduced the incidence of disease caused by *M. bovis* in both cattle and humans. In developing countries, however, animal TB is widely distributed, control measures are not applied or are applied sporadically, and pasteurization is rarely practiced. The direct correlation between *M. bovis* infection in cattle and disease in the human population has been well documented in industrialized countries. Whereas little information is available from developing countries (2,3), risk factors for *M. bovis* in both animals and humans are present in the tropics.

TB is a major opportunistic infection in HIV-infected persons (4). The vast majority of people carrying this dual infection live in developing countries; however, dual HIV and *M. bovis* infection has been reported in industrialized countries (5-11). The epidemic of HIV infection in developing countries, particularly countries in which *M. bovis* infection is present in animals and the conditions favor zoonotic transmission, could make zoonotic TB a serious public health threat to persons at risk (3,12-14).

We summarize available epidemiologic information on TB and zoonotic TB, examine risk factors that can influence the occurrence of zoonotic TB in developing countries, and describe the most recent TB activities of the World Health Organization (WHO) (15-18).

Address for correspondence: Ottorino Cosivi, Division of Emerging and Other Communicable Diseases, Surveillance and Control, CH-1211 Geneva 27, Switzerland; fax: 41-22-791-4893; e-mail: cosivio@who.ch.

### Human TB: Global Situation and Trends

The global incidence of TB is greatly underestimated. In 1995, 3.3 million cases were reported to the Global Tuberculosis Programme of WHO, whereas a more likely number is 8.8 million. Of the reported cases, 62% occurred in the Southeast Asian and Western Pacific regions, 16% in sub-Saharan Africa, and 7% to 8% in each of the regions of the Americas, Eastern Mediterranean, and Europe. Many countries, especially those with few resources, are unable to report all TB cases because of difficulties in identifying suspected cases, establishing a diagnosis, and recording and reporting cases.

In 1995, an estimated 8.8 million new TB cases occurred—5.5 million (62%) in the Southeast Asian and Western Pacific regions and 1.5 million (17%) in sub-Saharan Africa. The annual global incidence is predicted to increase to 10.2 million by the year 2000, an increase of 36% from 1990. Southeast Asia, Western Pacific regions, and sub-Saharan Africa will account for 81% of these new cases (Table 1). For 1990 to 1999, in the absence of effective control, global TB incidence and deaths will reach 88 million and 30 million, respectively (19); 70% of the new cases will occur in patients 15 to 59 years of age, the most economically productive segment of the population.

As a result of the HIV epidemic, the crude incidence rate of TB is expected to increase in sub-Saharan Africa from 191 cases per 100,000 in 1990 to 293 in 2000. However, the total number of new cases will double by the year 2000. Because of the HIV epidemic, the decline of the crude incidence rate in the Southeast Asian and Central and South American regions is expected to be slower than in previous years. In industrialized countries, a small increase in crude incidence rate and total cases is expected as the result of immigration from countries with a high prevalence of dual HIV and TB infection.

The worldwide incidence of HIV-attributable TB cases is estimated to increase from 315,000 (4% of the total TB cases) in 1990 to 1.4 million (14% of the total TB cases) by the year 2000. In 2000, approximately 40% of these HIV-attributable cases will occur in sub-Saharan Africa and 40% in Southeast Asia. Ten percent of the total number of TB cases expected during 1990 to 1999 are estimated to be attributable to HIV infection.

While demographic factors, such as population growth and changes in population structure, will largely account for the expected increase in TB incidence worldwide, the HIV epidemic in sub-Saharan Africa will have a greater role than demographic factors.

By the year 2000, 3.5 million persons will be dying of TB annually, an increase of 39% from 1990. In Southeast Asia alone, 1.4 million deaths will occur annually. During 1990 to 1999, an estimated 30 million will die of TB, with 9.7% of the cases attributable to HIV infection. *M. tuberculosis* will be largely responsible for the new TB

Table 1. Estimated human tuberculosis and HIV-attributable tuberculosis cases in 1990, 1995, and 2000, by region (19)

| | 1990 | | | 1995 | | | 2000 | | |
|---|---|---|---|---|---|---|---|---|---|
| Region | TB cases | Rate[a] | HIV-attributed | TB cases | Rate | HIV-attributed | TB cases | Rate | HIV-attributed |
| Southeast Asia | 3,106,000 | 237 | 66,000 | 3,499,000 | 241 | 251,000 | 3,952,000 | 247 | 571,000 |
| Western Pacific[b] | 1,839,000 | 136 | 19,000 | 2,045,000 | 140 | 31,000 | 2,255,000 | 144 | 68,000 |
| Africa | 992,000 | 191 | 194,000 | 1,467,000 | 242 | 380,000 | 2,079,000 | 293 | 604,000 |
| Eastern Mediterranean | 641,000 | 165 | 9,000 | 745,000 | 168 | 16,000 | 870,000 | 168 | 38,000 |
| Americas[c] | 569,000 | 127 | 20,000 | 606,000 | 123 | 45,000 | 645,000 | 120 | 97,000 |
| Eastern Europe[d] | 194,000 | 47 | 1,000 | 202,000 | 47 | 2,000 | 210,000 | 48 | 6,000 |
| Industrialized countries[e] | 196,000 | 23 | 6,000 | 204,000 | 23 | 13,000 | 211,000 | 24 | 26,000 |
| Total TB cases | 7,537,000 | 143 | 315,000 | 8,768,000 | 152 | 738,000 | 10,222,000 | 163 | 1,410,000 |
| Attributed to HIV | | | 4.2% | | | 8.4% | | | 13.8% |
| Increase since 1990 | | | | 16.3% | | | 35.6% | | |

[a]Rate: incidence of new cases per 100,000 population.
[b]Western Pacific Region of WHO except Japan, Australia, and New Zealand.
[c]American Region of WHO, except USA and Canada.
[d]Eastern European countries and independent states of the former USSR.
[e]Western Europe, USA, Canada, Japan, Australia, and New Zealand.

cases and deaths, but an unknown, and potentially important, proportion will be caused by *M. bovis*.

### Bovine TB in Developing Countries

Although prevalence data on animal TB in developing countries are generally scarce, information on bovine TB occurrence and control measures exists (20,21).

### Africa

Of 55 African countries, 25 reported sporadic/ low occurrence of bovine TB; six reported enzootic disease; two, Malawi and Mali, were described as having a high occurrence; four did not report the disease; and the remaining 18 countries did not have data (Figure 1).

Of all nations in Africa, only seven apply disease control measures as part of a test-and-slaughter policy and consider bovine TB a notifiable disease; the remaining 48 control the disease inadequately or not at all (Figure 2). Almost 15% of the cattle population are found in countries where bovine TB is notifiable and a test-and-slaughter policy is used. Thus, approximately 85% of the cattle and 82% of the human population of Africa are in areas where bovine TB is either partly controlled or not controlled at all.

### Asia

Of 36 Asian nations, 16 reported a sporadic/ low occurrence of bovine TB, and one (Bahrain) described the disease as enzootic; ten did not report bovine TB; and the remaining nine did not have data (Figure 3). Within the Asian region, seven countries apply disease control measures as part of a test-and-slaughter policy and consider bovine TB notifiable. In the remaining 29 countries, bovine TB is partly controlled or not controlled at all (Figure 4).

Of the total Asian cattle and buffalo populations, 6% and less than 1%, respectively, are found in countries where bovine TB is notifiable and a test-and-slaughter policy is used; 94% of the cattle and more than 99% of the buffalo populations in Asia are either only partly controlled for bovine TB or not controlled at all. Thus, 94% of the human population lives in countries where cattle and buffaloes undergo no control or only limited control for bovine TB.

### Latin American and Caribbean Countries

Of 34 Latin American and Caribbean countries, 12 reported bovine TB as sporadic/low occurrence, seven reported it as enzootic, and one (Dominican Republic) described occurrence as

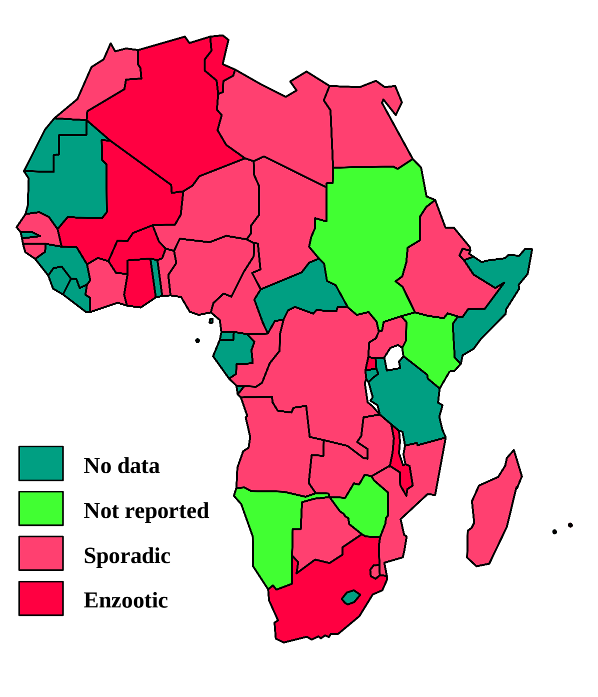

Figure 1. Bovine tuberculosis occurrence, Africa (21).

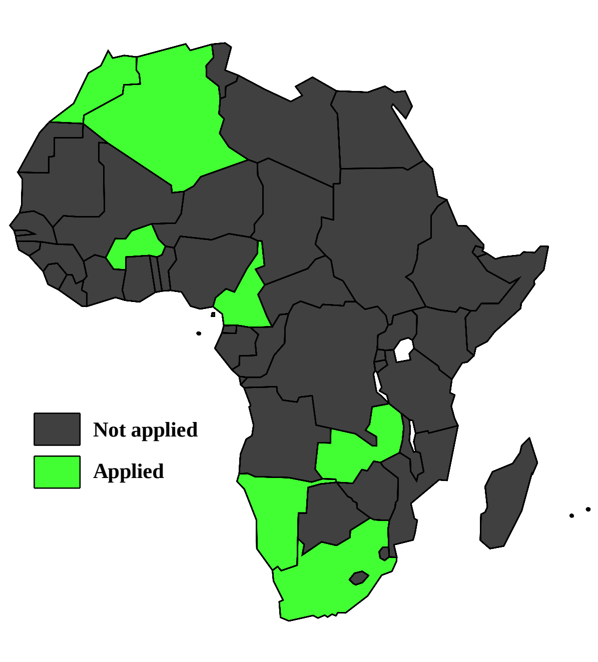

Figure 2. Control measures for bovine tuberculosis based on test-and-slaughter policy and disease notification, Africa (21).

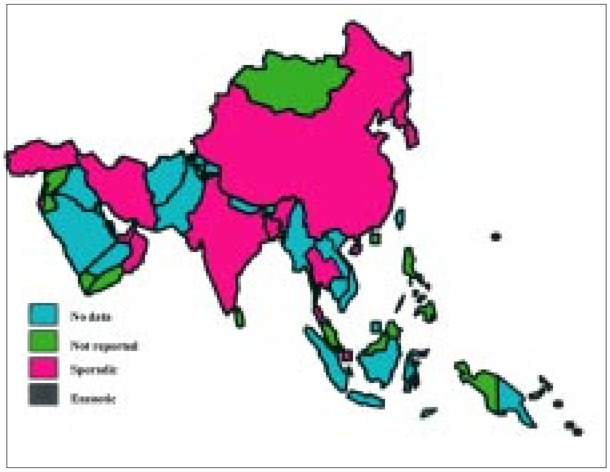

Figure 3. Bovine tuberculosis occurrence, Asia (21).

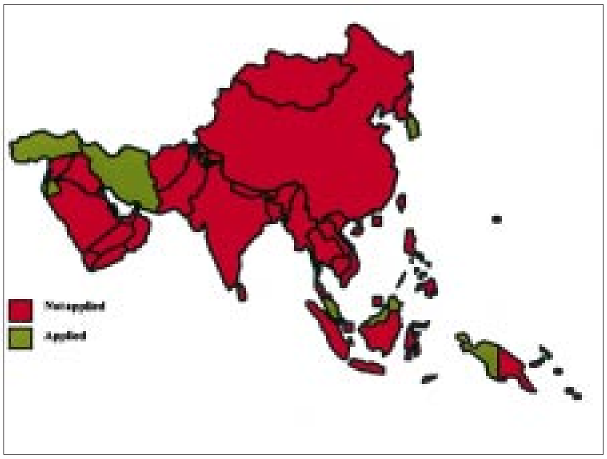

Figure 4. Control measures for bovine tuberculosis based on test-and-slaughter policy and disease notification, Asia (21).

high. Twelve countries did not report bovine TB. No data were available for the remaining two countries (Figure 5).

In the entire region, 12 countries apply disease control measures as part of a test-and-slaughter policy and consider bovine TB a notifiable disease. In the remaining 22 nations, the disease is partly controlled or not controlled at all (Figure 6). The regional prevalence of bovine TB has been estimated at 1% and higher in 67% of the total cattle population and 0.1% to 0.9% in a further 7%; the remaining 26% are free of the disease or are approaching the point of elimination (22).

Of the total Latin American and Caribbean cattle population, almost 76% is in countries where bovine TB is notifiable and a test-and-slaughter

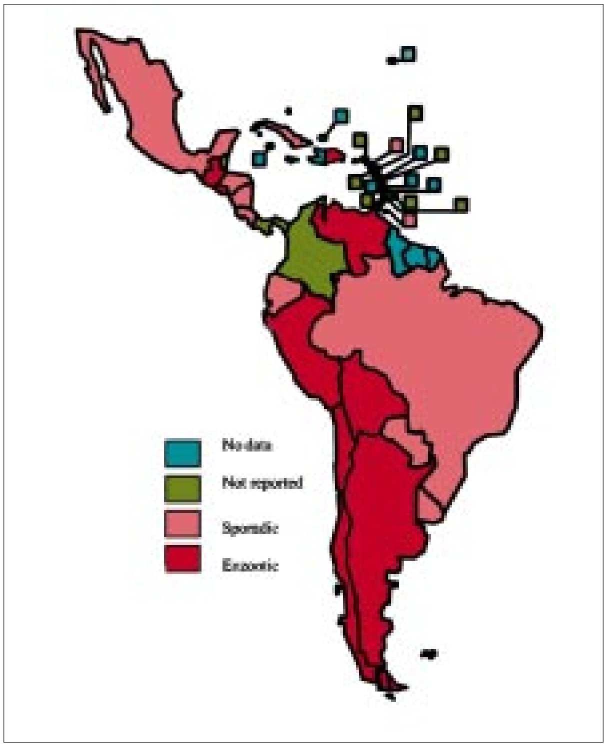

Figure 5. Bovine tuberculosis occurrence, Latin America and the Caribbean (21).

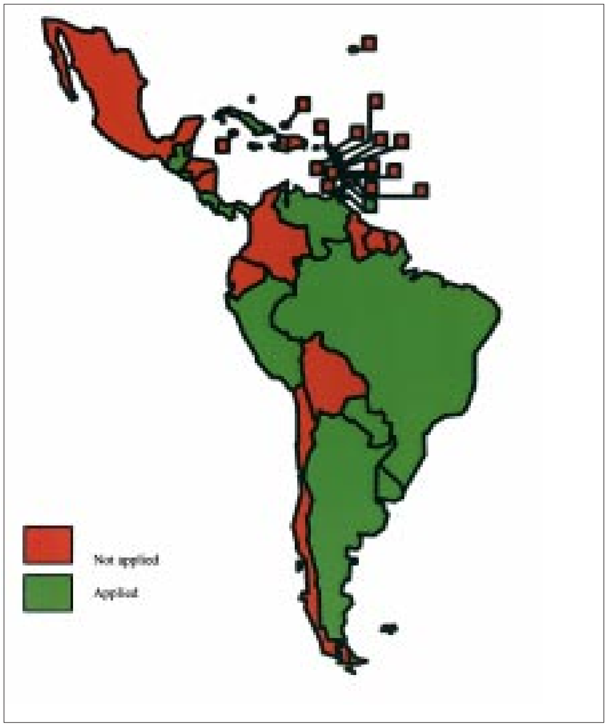

Figure 6. Control measures for bovine tuberculosis based on test-and-slaughter policy and disease notification, Latin America and the Caribbean (21).

policy is used. Thus, approximately 24% of the cattle population in this region is either only partly controlled for bovine TB or not controlled at all. It is also estimated that 60% of the human population live in countries where cattle undergo no control or only limited control for bovine TB.

### Zoonotic TB in Humans

TB caused by *M. bovis* is clinically indistinguishable from TB caused by *M. tuberculosis*. In countries where bovine TB is uncontrolled, most human cases occur in young persons and result from drinking or handling contaminated milk; cervical lymphadenopathy, intestinal lesions, chronic skin TB (lupus vulgaris), and other nonpulmonary forms are particularly common. Such cases may, however, also be caused by *M. tuberculosis*. Little is known of the relative frequency with which *M. bovis* causes nonpulmonary TB in developing nations because of limited laboratory facilities for the culture and typing of tubercle bacilli.

Agricultural workers may acquire the disease by inhaling cough spray from infected cattle; they develop typical pulmonary TB. Such patients may infect cattle, but evidence for human-to-human transmission is limited and anecdotal.

In regions where bovine TB has been largely eliminated, a few residual cases occur among elderly persons as a result of the reactivation of dormant lesions. These are fewer than 1% of all TB cases. Surveys in the United States, Scandinavia, and South England have shown that approximately half of these postprimary cases are pulmonary, a quarter involve the genitourinary tract (a rare occurrence in primary disease), and the remainder involve other nonpulmonary sites, notably cervical lymph nodes (23). In the same regions, approximately 10% of cases caused by *M. tuberculosis* are nonpulmonary, although, for reasons that are not clear, the incidence is higher, approximately 20%, in ethnic minority populations.

Information on human disease due to *M. bovis* in developed and developing countries is scarce. From a review of a number of zoonotic tuberculosis studies, published between 1954 and 1970 and carried out in various countries around the world, it was estimated that the proportion of human cases due to *M. bovis* acccounted for 3.1% of all forms of tuberculosis: 2.1% of pulmonary forms and 9.4% of extrapulmonary forms (24). Table 2 summarizes the findings of more recent reports of TB caused by *M. bovis* in industrialized countries.

Table 2. Human tuberculosis due to *Mycobacterium bovis*, industrialized countries

| Country (ref.) | Years | Cases No. | Cases % of total TB | Cases Pulmonary (% of total *M. bovis*) |
|---|---|---|---|---|
| Australia (25) | 1970-94 | 240 | 0.43-3.1 | 71.6[a] |
| England (23) | 1977-90 | 232 | 1.2 | 40.0 |
| Germany (26) | 1975-80 | 236 | 4.5 | 73.7 |
| Ireland | | | | |
| Rural (27) | 1986-90 | 17 | 6.4 | 70.6 |
| Urban (28) | 1982-85 | 9 | 0.9 | 88.8 |
| New Zealand (29) | 1983-90 | 22 | 7.2 | 31.8 |
| Spain (30) | 1986-90 | 10 | 0.9 | 50.0 |
| Sweden (17) | 1983-92 | 96 | 2.0 | - |
| Switzerland (31) | 1994 | 18 | 2.6 | - |
| U.S. (32) | 1954-68 | 6 | 0.3 | 33.3 |
| U.S. (9) | 1980-91 | 73 | 3.0 | 52.0[b]<br>12.0[c] |

[a] Overall percentage includes 80.6 % males and 51.2% females.
[b]Adults.
[c]Children.

Human disease caused by *M. bovis* has been confirmed in African countries. In an investigation by two Egyptian health centers, the proportions of sputum-positive TB patients infected with *M. bovis*, recorded during three observations, were 0.4%, 6.4%, and 5.4% (33). In another study in Egypt, nine of 20 randomly selected patients with TB peritonitis were infected with *M. bovis*, and the remaining with *M. tuberculosis* (34).

Isolation of *M. bovis* from sputum samples of patients with pulmonary TB has also been reported from Nigeria. Of 102 *M. tuberculosis* complex isolates, 4 (3.9%) were *M. bovis* (35). Another study in Nigeria reported that one of 10 mycobacteria isolated from sputum-positive cultures was *M. bovis* (36).

In a Zaire study, *M. bovis* was isolated from gastric secretions in two of five patients with pulmonary TB (37). In the same study, the prevalence of the disease in local cattle was approximately 8% by tuberculin testing and isolation of *M. bovis*.

In a recent investigation in Tanzania, seven of 19 lymph node biopsies from suspected extrapulmonary TB patients were infected with *M. tuberculosis* and four with *M. bovis* (14). No mycobacteria were cultured from the remaining

Table 3. Isolates from suspected extrapulmonary tuberculosis patients, Tanzania, 1994 (14)

| Occupation | No. of samples | *M. tuberculosis* | *M. bovis* | Neg. |
|---|---|---|---|---|
| Livestock keeper | 4 | 0 | 2 | 2 |
| Farmers | 6 | 2 | 1 | 3 |
| Children | 3 | 2 | 1 | 0 |
| Unknown | 6 | 3 | 0 | 3 |
| Total | 19 | 7 | 4 | 8 |

eight (Table 3). Although the number of samples was low, the high proportion (36%) of *M. bovis* isolates is of serious concern.

In an epidemiologic study in Zambia (38), an association between tuberculin-positive cattle and human TB was found. Households that reported a TB case within the previous 12 months were approximately seven times more likely to own herds containing tuberculin-positive cattle (odds ratio = 7.6; p = 0.004). Although this could be explained by zoonotic TB transmission, other factors such as transient sensitivity to tuberculin of cattle exposed to TB patients and coincidental environmental factors favoring both human clinical TB and sensitivity to bovine tuberculin should also be considered.

In Latin America, a conservative estimate would be that 2% of the total pulmonary TB cases and 8% of extrapulmonary TB cases are caused by *M. bovis*. These cases would therefore account for 7,000 new TB cases per year, a rate of nearly 2 per 100,000 inhabitants. From a nationwide study in Argentina during 1982 to 1984, 36 (0.47%) of 7,672 mycobacteria cultured from sputum samples were *M. bovis* (39). However, in another study in Santa Fe province (where most of the dairy cattle industry is concentrated) during 1984 to 1989, *M. bovis* caused 0.7% to 6.2% of TB cases (40).

Very limited data on the zoonotic aspects of *M. bovis* are available from Asian countries. However, cases of TB caused by *M. bovis* were not reported in early investigations in India (41).

### Epidemiology

Much information on the epidemiologic patterns of zoonotic TB has been obtained in this century from industrialized countries. However, some striking epidemiologic differences related to both animal and human populations in developing countries require particular attention.

### Risk Factors: Animal Population

**Animal reservoirs**. The widespread distribution of *M. bovis* in farm and wild animal populations represents a large reservoir of this microorganism. The spread of the infection from affected to susceptible animals in both industrialized and developing countries is most likely to occur when wild and domesticated animals share pasture or territory (42). Well-documented examples of such spread include infection in badgers (*Meles meles*) in the United Kingdom and possums (*Trichosurus vulpecula*) in New Zealand. Wild animal TB represents a permanent reservoir of infection and poses a serious threat to control and elimination programs.

**Milk production and animal husbandry.** Milk production has increased in most developing countries as a consequence of greater demand for milk for human consumption (43; Figure 7). This increased demand for milk—estimated at 2.5% per year for 1970 to 1988 for sub-Saharan Africa (44)—led to increases in the number of productive animals and milk imports and intensification of animal production through the introduction of more productive exotic breeds.

Although the prevalence of the disease within a country varies from area to area, the highest incidence of bovine TB is generally observed

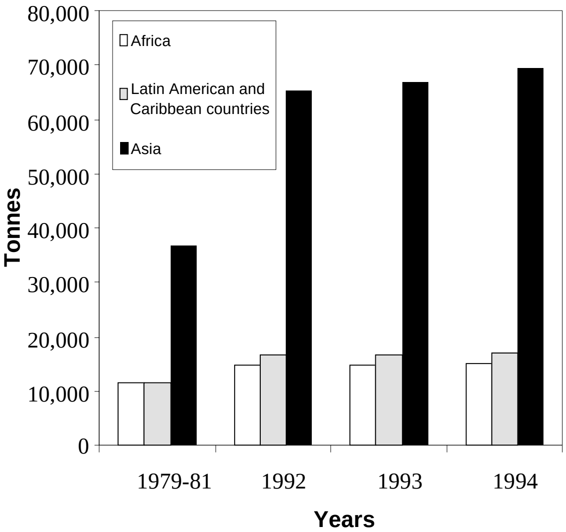

Figure 7. Cow milk production by region (43).

where intensive dairy production is most common, notably in the milksheds of larger cities (1). This problem is exacerbated where there is inadequate veterinary supervision, as is the case in most developing countries. In addition, in some industrialized countries such as the United States, where bovine TB is close to elimination, large dairy herds (i.e., 5,000 or more cows) that are crowded together represent the main source of infection (45).

In developing countries, bovine TB infects a higher proportion of exotic dairy breeds (*Bos taurus*) than indigenous zebu cattle (*Bos indicus*) and crossbred beef cattle (1). However, under intensive feedlot conditions, a death rate of 60% and depression of growth have been found in tuberculous zebu cattle (46). In those areas where extensive management is more common, animal crowding (e.g., near watering ponds, dip tanks, markets, and corrals) still plays a major role in the spread of the disease.

**Control measures and programs.** The basic strategies required for control and elimination of bovine TB are well known and well defined (47). However, because of financial constraints, scarcity of trained professionals, lack of political will, as well as the underestimation of the importance of zoonotic TB in both the animal and public health sectors by national governments and donor agencies, control measures are not applied or are applied inadequately in most developing countries.

Successful conduct of a test-and-slaughter policy requires sustained cooperation of national and private veterinary services, meat inspectors, and farmers, as well as adequate compensation for services rendered. Only a few developing countries can adhere to these requirements.

In addition, bovine TB does not often justify the emergency measures required for other zoonotic diseases (e.g., Rinderpest, East Coast fever, and foot and mouth disease). The full economic implications of zoonotic TB are, however, overlooked in many developing nations where the overall impact of the disease on human health and animal production needs to be assessed. According to recent estimates, annual economic loss to bovine TB in Argentina is approximately 63 million US dollars (48). In a study recently conducted in Turkey, the estimated socioeconomic impact of bovine TB to both the agriculture and health sectors was approximately 15 to 59 million US dollars per year (49).

Several Latin American countries, through agreements between governments and cattle owners associations, have made the decision to control and eliminate bovine TB. Where foot and mouth disease has been eliminated, bovine TB and other existing infections such as brucellosis become important because of their impact on the meat and live animal export trade. Bovine TB and brucellosis also limit the development of the dairy industry and its expansion at the regional level.

### Risk Factors: Human Population

**Close physical contact.** Close physical contact between humans and potentially infected animals is present in some communities, especially in developing regions. For example, in many African countries cattle are an integral part of human social life; they represent wealth and are at the center of many events and, therefore, gatherings. In addition, with 65% of African, 70% of Asian, and 26% of Latin American and Caribbean populations working in agriculture, a significant proportion of the population of these regions may be at risk for bovine TB.

**Food hygiene practices.** Consumption of milk contaminated by *M. bovis* has long been regarded as the principal mode of TB transmission from animals to humans (1). In regions where bovine TB is common and uncontrolled, milkborne infection is the principal cause of cervical lymphadenopathy (scrofula) and abdominal and other forms of nonpulmonary TB. Although proper food hygiene practices could play a major role in controlling these forms of TB, such practices are often difficult to institute in developing countries.

In all countries of sub-Saharan Africa, there is active competition between large-scale, often state-run, processing and marketing enterprises and the informal sector. The informal sector can ignore standards of hygiene and quality, and producers often sell directly to the final consumers. In addition, an estimated 90% of the total milk produced is consumed fresh or soured (44). Although it has been stated that Africans generally boil milk and that the souring process destroys *M. bovis* (44), other sources strongly contradict these statements (39). *M. bovis* was isolated from seven (2.9%) of 241 samples of raw milk in Ethiopia (17). Both *M. bovis* and *M. tuberculosis* have also been found in milk samples in Nigeria (36) and Egypt (34). Thus, serious public health implications of potentially contaminated milk and milk products should not be underestimated.

**HIV/AIDS.** According to recent WHO global estimates, of the 9.4 million people infected with both HIV and TB in mid-1996, 6.6 million (70%) live in sub-Saharan Africa (4). The greatest impact of HIV infection on TB is in populations with a high prevalence of TB infection among young adults. The occurrence of both infections in one person makes TB infection very likely to progress to active disease.

In many developing countries, TB is the most frequent opportunistic disease associated with HIV infection. HIV seroprevalence rates greater than 60% have been found in TB patients in various African countries (4). Persons infected with both pathogens have an annual risk of progression to active TB of 5% to 15%, depending on their level of immunosuppression; approximately 10% of non-HIV infected persons newly infected with TB become ill at some time during their lives. In the remaining 90%, effective host defenses prevent progression from infection to disease.

TB cases due to *M. bovis* in HIV-positive persons also resemble disease caused by *M. tuberculosis*. Thus, they manifest as pulmonary disease, lymphadenopathy, or, in the more profoundly immunosuppressed, disseminated disease.

*M. bovis* has been isolated from HIV-infected persons in industrialized countries. In France, *M. bovis* infection accounted for 1.6% of TB cases in HIV-positive patients. All isolated strains were resistant to isoniazid (7). Taking into consideration the intrinsic resistance of *M. bovis* to pyrazinamide, two of the first-line anti-TB drugs were not effective. WHO-recommended standard treatment for new TB cases includes, in the initial phase, isoniazid, rifampicin, pyrazinamide, and streptomycin or ethambutol. In situations of high primary resistance to isoniazid and streptomycin, the intrinsic resistance of *M. bovis* to pyrazinamide may severely limit the efficacy of treatment of TB caused by *M. bovis*.

In a Paris hospital, a source patient with pulmonary TB due to a multidrug-resistant strain of *M. bovis* led to active disease in five patients. Disease occurred 3 to 10 months after infection (10). This observation led to three concerns: 1) human-to-human *M. bovis* transmission leading to overt disease, 2) a short interval between infection and overt disease, and 3) disseminated multidrug-resistant *M. bovis*.

In another study, conducted in San Diego, California, one of 24 adults with pulmonary TB and 11 of 24 adults with nonpulmonary TB due to *M. bovis* had AIDS. One of 25 children, a 16-year-old boy with abdominal TB, was also HIV-positive (9).

It is commonly believed that *M. bovis* is less virulent than *M. tuberculosis* in humans and therefore less likely to lead to overt postprimary disease and that human-to-human transmission leading to infectious disease is rare. However, if the apparent difference in virulence is the result of differences in responsiveness of the host defense mechanisms, HIV-induced immunosuppression could well lower host defenses leading to overt disease after infection.

### Surveillance of TB due to *M. bovis*

The use of direct smear microscopy as the only method for diagnosis of suspected TB, although an essential requirement of any national TB program, could partly explain the relatively low notification rate of disease caused by *M. bovis* in developing countries. Direct smear microscopy does not permit differentiation between species of the *M. tuberculosis* complex; in addition, culture and speciation are often not carried out, and even when culture facilities are available, *M. bovis* grows poorly in standard Löwenstein-Jensen medium, one of the most widely used culture media (50). In some countries, human disease caused by *M. bovis* is merely reported as TB to avoid inquiries from disease control agencies, which might generate problems of patient confidentiality (2).

The collection of representative data on the incidence of TB due to *M. bovis* from most laboratories in developing countries has additional problems. For example, the location and coverage of laboratories are often biased towards city populations; sputum specimens may predominate, with relatively few specimens from extrapulmonary lesions, particularly among children. Specimens from children with TB are frequently negative on culture, and biopsies are difficult to take from lesions.

Recent outbreaks of multidrug-resistant TB in some parts of the world underscore the need for surveillance through wider application of reliable culture and drug susceptibility tests.

### Control Measures and Programs in Developing Countries

Bovine TB can be eliminated from a country or region by implementing a test-and-slaughter policy, if no other reservoir host of infection exists. While the test-and-slaughter policy is likely to remain the backbone of national elimination bovine TB programs, the policy has numerous constraints in developing countries. Alternative strategies (e.g., programs based on slaughterhouse surveillance and traceback of tuberculous animals to herds of origin) may be technically and economically more appropriate in these countries.

Measures to prevent transmission of infection should be the primary objective to be achieved with trained public health personnel, public education, and proper hygienic practices. Test-and-slaughter programs may be feasible and appropriate in areas with low bovine TB prevalence and effective control of animal movement.

### Animal Vaccination and Research Developments

Although not usually considered relevant to elimination programs in livestock (47), vaccination of animals against TB would be a viable strategy in two disease control situations: in domesticated animals in developing countries and in wildlife and feral reservoirs of disease in industrialized countries where test-and-slaughter programs have failed to achieve elimination of the disease.

Many issues need to be addressed before vaccination becomes a realistic option for control of disease in cattle and other animals. First, a highly effective vaccine needs to be developed. The results obtained globally with bacillus Calmette-Guérin (BCG) have been suboptimal, and efficacy has varied considerably from region to region (42,51). Secondly, the delivery of the vaccine poses few problems in domesticated animals, but it is fraught with difficulties in wild animals. Thirdly, vaccination may compromise diagnostic tests. A vaccine that induces tuberculin reactivity would invalidate the key diagnostic tool used in control programs. Fourthly, short of performing lengthy and expansive field studies, evaluation of the protective efficacy of a new vaccine will pose serious difficulties. Traditionally, the guinea pig and mouse have been used for this purpose, but the information gained has been of little value. Recent work has, however, indicated that deer may well prove a suitable mammal for evaluating new vaccines and optimum delivery systems (52).

Enzyme-linked immunosorbent assay and gamma-interferon tests may prove to be more sensitive and specific than the tuberculin test and may facilitate diagnostic procedures. Nucleic acid-based technology, notably polymerase chain reaction and related methods, may provide more rapid, sensitive, and specific diagnostic tools. Multicenter studies of the applicability of these techniques to the diagnosis of human TB have, however, shown that their sensitivity and specificity are not as high as originally expected and that many problems need to be solved before the techniques are introduced into routine laboratory practice (53). Restriction fragment length polymorphism analysis (DNA fingerprinting) could be useful in epidemiologic studies that trace the spread of disease between cattle, other animals, and humans (54) or in the rapid differentiation of *M. bovis* within the *M. tuberculosis* complex (55). The use of these techniques is limited by resources in most developing countries.

### WHO and Zoonotic TB

The public health importance of animal TB was recognized early by WHO, which in its 1950 report of the Expert Committee on Tuberculosis (56) stated: "The committee recognizes the seriousness of human infection with bovine tuberculosis in countries where the disease in cattle is prevalent. There is the danger of transmission of infection by direct contact between diseased cattle and farm workers and their families, as well as from infected food products." Since then, TB in animals has been controlled and almost eliminated in several industrialized countries but in very few developing countries.

More recently, WHO has been involved in zoonotic TB through the activities of the Division of Emerging and other Communicable Diseases Surveillance and Control at WHO in Geneva (WHO/EMC) and the Veterinary Public Health program of the WHO Regional Office for the Americas, Pan American Health Organization (PAHO/HCV).

WHO/EMC has organized and coordinated a working group of experts from countries worldwide (15-17). Their subjects are epidemiology, public health aspects, control, and research on zoonotic TB. In addition, a joint WHO, Food and Agriculture Organization of the United Nations (FAO), and Office International des Epizooties (OIE) Consultation on Animal Tuberculosis Vaccines was held to review current knowledge on TB vaccine development for humans and animals and make recommendations for animal TB vaccine research and development (57). Promising results of cattle vaccination with low doses of BCG were reported. It is also planned for field trial cattle vaccination to commence early in 1998 in Madagascar in collaboration with national and international research institutions, OIE and WHO. In the framework of the working group activities, the guidelines for speciation within the *Mycobacterium tuberculosis* complex (50) have been prepared to respond to the growing need for reliable differentiation between *M. tuberculosis*, *M. africanum*, and *M. bovis* and to promote and strengthen surveillance.

A Plan of Action for the Eradication of Bovine Tuberculosis in the Americas (18) has been developed by PAHO in collaboration with member countries of the region. PAHO/HCV, in cooperation with the Pan American Institute for Food Protection and Zoonosis (INPPAZ), Buenos Aires, Argentina, and other technical institutions (e.g., FAO), provides technical support to the regional plan. PAHO/HCV activities train specialists in diagnosis, reporting, surveillance systems, and quality control of reagents, as well as supporting the planning and implementation of national programs. INPPAZ acts as a reference center for these activities. The first phase of the regional plan is expected to lead, in the next 10 years, to the elimination of bovine TB from countries with more advanced national programs. In the remaining countries, the objectives will be to strengthen epidemiologic surveillance, defining areas at risk and setting up control and elimination programs.

### Conclusions

Although the epidemiology of bovine TB is well understood and effective control and elimination strategies have been known for a long time, the disease is still widely distributed and often neglected in most developing countries. Its public health consequences, although well documented from the past experiences of industrialized countries, have scarcely been investigated and are still largely ignored in these regions. Because of the animal and public health consequences of *M. bovis*, disease surveillance programs in humans should be considered a priority, especially in areas where risk factors are present. The increase of TB in such areas calls for stronger intersectoral collaboration between the medical and veterinary professions to assess and evaluate the scale of the problem, mostly when zoonotic TB could represent a significant risk, for example, in rural communities and in the workplace.

Industrialized countries, where the test-and-slaughter policies have not completely eliminated infection in cattle because of wild animal reservoirs, are now reconsidering wild animal vaccination. Any vaccination research and development program should therefore also take into account the possible application of vaccines to cattle, particularly in developing countries.

In developing countries, where HIV and bovine TB are likely to be common, particularly in young persons, the ability of HIV infection to abrogate any host factors that prevent the progression of infection by *M. bovis* to overt disease may lead to higher incidence and case-fatality rates for human TB caused by this species and increased human-to-human transmission of this disease. This should be of great concern in those developing countries where bovine TB is present and measures to control spread of infection are not applied or are applied inadequately. Research is needed to determine when *M. bovis* is of zoonotic importance and what the underlying mechanisms of transmission are. Locally operative risk factors for zoonotic TB should therefore be identified to determine persons at risk and develop appropriate control measures. International cooperation in all aspects of zoonotic TB remains essential in the fight against this disease.

### References

1. Acha PN, Szyfres B. Zoonotic tuberculosis. In: Zoonoses and communicable diseases common to man and animals. 2nd edition. Washington: Pan American Health Organization/World Health Organization; 1987: Scientific Publication No. 503.
2. Collins CH, Grange JM. A review. The bovine tubercle bacillus. J Appl Bacteriol 1983;55:13-29.

3. Cosivi O, Meslin F-X, Daborn CJ, Grange JM. The epidemiology of *Mycobacterium bovis* infection in animals and humans, with particular reference to Africa. Scientific and Technical Review 1995;14:733-46.
4. Raviglione MC, Snider DE, Kochi A. Global epidemiology of tuberculosis. JAMA 1995;273:220-6.
5. Houde C, Dery P. *Mycobacterium bovis* sepsis in an infant with human immunodeficiency virus infection. Pediatr Infect Dis J 1988;7:810-2.
6. Cornuz J, Fitting JW, Beer V, Chave JP. *Mycobacterium bovis* and AIDS. AIDS 1991;5:1038-9.
7. Dupon M, Ragnaud JM. Tuberculosis in patients infected with human immunodeficiency virus 1. A retrospective multicentre study of 123 cases in France. Quarterly Journal of Medicine [New Series 85] 1992;306:719-30.
8. Yates MD, Pozniak A, Grange JM. Isolation of mycobacteria from patients seropositive for the human immunodeficiency virus (HIV) in south east England: 1984-92. Thorax 1993;48:990-5.
9. Dankner WM, Waecker NJ, Essey MA, Moser K, Thompson M, Davis CH. *Mycobacterium bovis* infections in San Diego: a clinicoepidemiologic study of 73 patients and a historical review of a forgotten pathogen. Medicine (Baltimore) 1993;72:11-37.
10. Bouvet E, Casalino E, Mendoza-Sassi G, Lariven S, Vallee E, Pernet M, et al. A nosocomial outbreak of multidrug-resistant *Mycobacterium bovis* among HIV-infected patients. A case-control study. AIDS 1993;7:1453-60.
11. Boletín Epidemiológico Semanal. Vigilancia de la tuberculosis zoonótica por *M. bovis*: sistema de enfermedades de declaración obligatoria 1982-1995. Red Nacional de Vigilancia Epidemiológica de España, Centro Nacional de Epidemiología 1995;3:183-4.
12. Grange JM, Daborn C, Cosivi O. HIV-related tuberculosis due to *Mycobacterium bovis*. Eur Respir J 1994;7:1564-6.
13. Moda G, Daborn CJ, Grange JM, Cosivi O. The zoonotic importance of *Mycobacterium bovis*. Tubercle and Lung Disease 1996;77:103-8.
14. Daborn CJ, Grange JM, Kazwala RR. The bovine tuberculosis cycle—an African perspective. J Appl Bacteriol [Symposium Supplement] 1997;81:27s-32s.
15. World Health Organization. Report of the WHO meeting on animal tuberculosis; 1992 Apr 27; Cairo, Egypt. Geneva: The Organization; 1992. Unpub. document WHO/CDS/VPH/92.112.
16. World Health Organization. Report of the WHO meeting on zoonotic tuberculosis (*Mycobacterium bovis*), with the participation of the FAO; 1993 Nov 15. Geneva, Switzerland. Geneva: The Organization; 1993. Unpub. document WHO/CDS/VPH/93.130.
17. World Health Organization. Report of WHO Working Group on zoonotic tuberculosis (*Mycobacterium bovis*), with the participation of FAO; 1994 June 14. Mainz, Germany. Geneva: The Organization; 1994. Unpub. document WHO/CDS/VPH/94.137.
18. World Health Organization/Pan American Health Organization. VIII Inter-American meeting, at the ministerial level on animal health; 1993 Apr 27-29. Washington, DC. Washington: The Organizations; 1993. Document RIMSA 8/26.
19. Dolin PJ, Raviglione MC, Kochi A. Global tuberculosis incidence and mortality during 1990-2000. Bull World Health Organ 1994;72:213-20.
20. Thoen CO, Steele JH, editors. Regional and Country Status Reports. Part 2. *Mycobacterium bovis* infection in animals and humans. Ames (IA): Iowa State University Press; 1995. p. 167-345.
21. Food and Agriculture Organization. In: Welte WR, editor. FAO/OIE/WHO Animal Health Yearbook 1993. Washington: The Organization; 1994. FAO Animal Production and Health Series No. 33.
22. de Kantor IN, Ritacco V. Bovine tuberculosis in Latin America and the Caribbean: current status, control and eradication programs. Vet Microbiol 1994;40:5-14.
23. Grange JM, Yates MD. Zoonotic aspects of *Mycobacterium bovis* infection. Vet Microbiol 1994;40:137-51.
24. Gervois M, Vaillant JM, Fontaine JF, Laroche G, Dubois G. Epidemiologie de l'infection humaine par *Mycobacterium bovis*. Archivio Monaldi per la tisiologia e le malattie dell'apparato respiratorio 1972;27:294-317.
25. Cousins DV, Williams SN. A study of *Mycobacterium bovis* infection in Australian patients 1970-1994. In: Griffit F, de Lisle G, editors. Tuberculosis in wildlife and domestic animals. Otago, New Zealand: University of Otago Press: 1995. p. 260-3.
26. Krebs A, Käppler W. Die Bedeutung von *Mycobacterium bovis* in der Tuberkuloseepidemiologie. Zeitschift für Erkrankungen der Atmungs Organe 1982;158:101-9.
27. Cormican MG, Flynn J. Tuberculosis in the West of Ireland 1986-1990. Ir J Med Sci 1992;161:70-2.
28. Collins C, Kelly P, Bryne C, Denham F, Clancy L. Is bovine, atypical or resistant tuberculosis a problem? Ir Med J 1987;80:66-7.
29. Brett JL, Humble MW. Incidence of human tuberculosis caused by *Mycobacterium bovis*. N Z Med J 1991;104:13-4.
30. Sauret J, Jolis R, Ausina V, Castro E, Cornudella R. Human tuberculosis due to *Mycobacterium bovis*: report of 10 cases. Tubercle and Lung Disease 1992;73:388-91.
31. La tuberculose en Suisse en 1994. Bern, Switzerland: Bulletin 37, Office fédéral de la santé publique;1994. p.10-1.
32. Karlson AG, Carr DT. Tuberculosis caused by *Mycobacterium bovis*. Report of six cases: 1954-1968. Ann Intern Med 1970;73:979-83.
33. Elsabban MS, Lofty O, Awad WM, Soufi HS, Mikhail DG, Hammam HM, et al. Bovine tuberculosis and its extent of spread as a source of infection to man and animals in Arab Republic of Egypt. In: Proceedings of the International Union Against Tuberculosis and Lung Disease Conference on Animal Tuberculosis in Africa and the Middle East; 1992 Apr 28-30; Cairo, Egypt. Paris: The Union; 1992. p.198-211.
34. Nafeh MA, Medhat A, Abdul-Hameed A-G, Ahmad YA, Rashwan NM, Strickland GT. Tuberculous peritonitis in Egypt: the value of laparoscopy in diagnosis. Am J Trop Med Hyg 1992;47:470-7.
35. Idigbe EO, Anyiwo CE, Onwujekwe DI. Human pulmonary infections with bovine and atypical mycobacteria in Lagos, Nigeria. Journal of Tropical Medicine and Hygiene 1986;89:143-8.

36. Idrisu A, Schnurrenberger P. Public health significance of bovine tuberculosis in four northern states of Nigeria: a mycobacteriologic study. Nigerian Medical Journal 1977;7:384-7.
37. Mposhy M, Binemo-Madi C, Mudakikwa B. Incidence de la tuberculose bovine sur la santé des populations du Nord-Kivu (Zaïre). Rev Elev Med Vet Pays Trop 1983;36:15-8.
38. Cook AJC, Tuchili LM, Buve A, Foster SD, Godfrey-Faussett P, Pandey GS, McAdam KPWJ. Human and bovine tuberculosis in the Monze district of Zambia—a cross-sectional study. Br Vet J 1996;152:37-46.
39. Barrera L, de Kantor IN. Nontuberculous mycobacteria and *Mycobacterium bovis* as a cause of human disease in Argentina. Tropical and Geographical Medicine 1987;39:222-7.
40. Sequeira de Latini MD, Latini OA, Lopez ML, Cecconi JO. Tuberculosis bovina en seres humanos. 2a. parte: Periodo 1977-1989. Revista Argentina del Torax 1990;51:13-1.
41. Lall JM. Tuberculosis among animals in India. The Veterinary Bulletin 1969;39:385-90.
42. O'Reilly LM, Daborn CJ. The epidemiology of *Mycobacterium bovis* infections in animals and man: a review. Tubercle and Lung Disease 1995;76(Suppl 1):1-46.
43. Food and Agriculture Organization. Production. Vol. 47. Rome: Food and Agriculture Organization of the United Nations; 1993. FAO Statistic Series No. 117.
44. Walshe MJ, Grindle J, Nell A, Bachmann M. Dairy development in sub-Saharian Africa. Washington DC: World Bank, 1991. African Technical Department Series; World Bank Technical Paper No. 135.
45. National Research Council. Livestock disease eradication—evaluation of the cooperative State-Federal Bovine Tuberculosis Eradication Program. Washington: National Academy Press; 1994.
46. Blancou J, Cheneau Y. Influence de la tuberculose sur le gain de poids de zébus à l'engrais. Rev Elev Med Vet Pays Trop 1974;27:75-80.
47. World Health Organization. Third Report of the Joint FAO/WHO Expert Committee on Zoonoses. Geneva: The Organization; 1967. Technical Report Series No. 378.
48. Maggi C, Alvarez E, de Kantor I, Nader A. Methodology for estimating the losses caused by bovine tuberculosis in Argentina. Scientific and Technical Review. In press 1998.
49. Barwinek F, Taylor NM. Assessment of the socio-economic importance of bovine tuberculosis in Turkey and possible strategies for control or eradication. Bakanliklar, Ankara, Turkey: Turkish-German Animal Health Information Project, General Directorate of Protection and Control; 1996.
50. Grange JM, Yates MD, de Kantor I. Guidelines for speciation within the *Mycobacterium tuberculosis* complex. 2nd ed. Geneva: World Health Organization; 1996. Unpub. document WHO/EMC/ZOO/96.4.
51. Malin AS, Young DB. Designing a vaccine for tuberculosis. Unraveling the tuberculosis genome—can we build a better BCG? Br Med J 1996;312:1495.
52. Griffin JFT, Mackintosh CG, Buchan GS. Animal models of protective immunity in tuberculosis to evaluate candidate vaccines. Trends Microbiol 1995;3:418-24.
53. Doucet-Populaire F, Lalande V, Carpentier E, Bourgoin A, Dailloux M, Bollet A, et al. A blind study of the polymerase chain reaction for the detection of *Mycobacterium tuberculosis* DNA. Tubercle and Lung Disease 1996;77:358-62.
54. van Embden JDA, Schouls LM, van Soolingen D. Molecular techniques: application in epidemiologic studies. In: Thoen CO, Steele JH, editors. *Mycobacterium bovis* infection in animals and humans. Ames (IA): Iowa State University Press; 1995. p. 15-27.
55. Liebana E, Aranaz A, Francis B, Cousins D. Assessment of genetic markers for species differentiation within the *Mycobacterium tuberculosis* complex. J Clin Microbiol 1996;34:933-8.
56. World Health Organization. Expert Committee on Tuberculosis report on the Fifth Session; 1950 Sep 11-16; Geneva, Switzerland. Geneva: The Organization; 1951. Technical Report Series 32.
57. World Health Organization. Report of a WHO/FAO/OIE consultation on animal tuberculosis vaccines; 1994 Aug 3-5; Geneva, Switzerland. Geneva: The Organization; 1994. Unpub. document WHO/CDS/VPH/94.138.
